# Differences in Ventilation Management and Outcomes between the Two First Waves of the COVID-19 Pandemic—A Comparison between Two Nationwide Observational Studies in The Netherlands

**DOI:** 10.3390/jcm12134507

**Published:** 2023-07-05

**Authors:** Liselotte Hol, Marcus J. Schultz, Ignacio Martin-Loeches, David M. P. van Meenen, Ary Serpa Neto, Frederique Paulus, PRoAcT-COVID Investigators

**Affiliations:** 1 Department of Anesthesiology, Amsterdam University Medical Centers, Location AMC, 1105 AZ Amsterdam, The Netherlands; 2 Department of Intensive Care, Amsterdam University Medical Centers, Location AMC, 1105 AZ Amsterdam, The Netherlands; 3 Mahidol Oxford Tropical Medicine Research Unit (MORU), Mahidol University, Bangkok 10400, Thailand; 4 Nuffield Department of Medicine, University of Oxford, Oxford OX37BN, UK; 5 Department of Intensive Care Medicine, Multidisciplinary Intensive Care Research Organization (MICRO), St James’s Street, Leinster, D08 NYH1 Dublin, Ireland; 6 Department of Clinical Medicine, Trinity College, D02 PN40 Dublin, Ireland; 7 Department of Critical Care Medicine, Hospital Israelite Albert Einstein, Sao Paolo 05652-900, Brazil; 8 Department of Critical Care Medicine, Austin Hospital and University of Melbourne, Melbourne 3084, Australia; 9 Centre of Applied Research, Faculty of Health, Amsterdam University of Applied Sciences, 1091 GC Amsterdam, The Netherlands

**Keywords:** coronavirus disease 2019, COVID-19, invasive ventilation, ventilation management, complications

## Abstract

The aim of this analysis was to compare ventilation management and outcomes in invasively ventilated patients with acute hypoxemic respiratory failure due to coronavirus disease 2019 (COVID-19) between the first and second wave in the Netherlands. This is a post hoc analysis of two nationwide observational COVID-19 studies conducted in quick succession. The primary endpoint was ventilation management. Secondary endpoints were tracheostomy use, duration of ventilation, intensive care unit (ICU) and hospital length of stay (LOS), and mortality. We used propensity score matching to control for observed confounding factors. This analysis included 1122 patients from the first and 568 patients from the second wave. Patients in the second wave were sicker, had more comorbidities, and had worse oxygenation parameters. They were ventilated with lower positive end-expiratory pressure and higher fraction inspired oxygen, had a lower oxygen saturation, received neuromuscular blockade more often, and were less often tracheostomized. Duration of ventilation was shorter, but mortality rates were similar. After matching, the fraction of inspired oxygen was lower in the second wave. In patients with acute hypoxemic respiratory failure due to COVID-19, aspects of respiratory care and outcomes rapidly changed over the successive waves.

## 1. Introduction

The recurrent pandemic of coronavirus disease 2019 (COVID-19) has resulted in a tremendous health burden [1]. At the time of writing, more than 631 million confirmed COVID-19 cases and 6.6 million casualties have been reported worldwide [1]. Most, if not all, countries, including the Netherlands, have endured several waves of COVID-19 outbreaks, during which many patients with acute hypoxemic respiratory failure needed admission to an intensive care unit (ICU) for escalation of respiratory support, mostly invasive ventilation. The often hectic situation did not stop the ICU communities from providing excellent care with each wave, even though most healthcare workers struggled with many uncertainties, including how best to provide respiratory support in the early stages of the pandemic.

Due to changing hospital and ICU admission policies, modifications in national and international guidelines that involved the introduction or abundance of certain therapies, the care and outcomes of critically ill COVID-19 patients may have changed over successive outbreaks. Indeed, reports from countries worldwide suggest variances in care and trends towards a shorter length of hospital stay and even lower mortality rates in consecutive waves [2,3,4,5,6,7,8,9]. To date, few reports have described changes in care and outcomes in invasively ventilated COVID-19 patients within a single center, region, or country.

The aim of this analysis was to compare ventilation management and outcomes in critically ill invasively ventilated COVID-19 patients between the first and the second wave of the outbreak in the Netherlands. For this, we used the datasets of two nation-wide multicenter studies, known as the ‘PRactice of VENTilation in patients with COVID-19’ (PRoVENT–COVID) [10] and the ‘Practice of Adjunctive Treatments in intensive care unit patients with COVID-19’ (PRoAcT-COVID) [11]. While PRoVENT–COVID enrolled patients in the first wave of the national outbreak, PRoAcT-COVID enrolled patients in the second wave, which started within three months after the end of the first wave. We hypothesized that there would be important differences with regard to ventilation management between patients included in these two studies, conducted in quick successions. We used propensity score matching to control for observed confounding factors that could have affected these outcomes.

## 2. Materials and Methods

### 2.1. Design, Ethics and Patients

This is a post hoc analysis of PRoVENT–COVID and PRoAcT-COVID, two nationwide, multicenter, observational studies conducted in critically ill COVID-19 patients during the first and second wave of the national outbreak in the Netherlands [10,11]. The Institutional Review Board of the Amsterdam University Medical Centers, location AMC, Amsterdam, the Netherlands, approved the study protocols of PRoVENT–COVID (7 April 2020; W20_157 # 20,171) and PRoAcT-COVID (11 December 2020; W20_526 # 20,583). For both studies, the requirement of written informed consent was waived as the studies were purely observational and only captured data that were already collected as part of standard care. The studies were registered at clinicaltrials.gov (study identifiers NCT04346342 and NCT04719182). PRoVENT–COVID enrolled patients between 1 March and 1 June 2020 in 22 ICUs. PRoAcT-COVID enrolled patients between 1 September 2020 and 1 January 2021 in 16 ICUs. Fourteen centers participated in both studies.

Patients were eligible for participation in PRoVENT–COVID if: (1) aged 18 years or older; (2) admitted to one of the ICUs of a participating hospital; and (3) receiving invasive ventilation for COVID-19 that was confirmed by RT–PCR for SARS-CoV-2. The inclusion criteria in PRoAcT-COVID were similar, with the exception that this second study also enrolled patients under other forms of respiratory support, i.e., noninvasive ventilation and high-flow nasal oxygen therapy. For this current analysis, we excluded patients from PRoAcT-COVID that did not receive invasive ventilation.

### 2.2. Data Collected

Trained data collectors captured baseline characteristics, including age, sex, weight, height, comorbidities, home medication, kidney function, severity of acute respiratory distress syndrome (ARDS), and the Simplified Acute Physiology Score (SAPS) II. Ventilation mode, positive end-expiratory pressure (PEEP), fraction of inspired oxygen (FiO_2_), and oxygen saturation by pulse oximetry (SpO_2_) were collected at a fixed time in the morning until day 4. We also captured use of prone positioning, extracorporeal membrane oxygenation (ECMO), and neuromuscular blockade. In addition, we collected tracheostomy use till day 28, the last day of invasive ventilation, the last day of stay in the ICU and hospital, and life status at ICU and hospital discharge as well as at day 28 and 90.

### 2.3. Study Endpoints

The primary endpoint was ventilation management, consisting of PEEP, FiO_2_, SpO_2_, and the use of prone positioning, ECMO, or neuromuscular blockade. Secondary endpoints included use of tracheostomy, duration of invasive ventilation, ICU and hospital LOS, and ICU, hospital, and 28- and 90-day mortality.

### 2.4. Definitions

ARDS was defined according to the current definition [12]. Prone positioning was defined as turning a patient from supine to a (semi) prone position because of refractory hypoxemia. Neuromuscular blockade was defined as (incidental or continuous) use of a neuromuscular blocking agent (NMBA), wherein use to facilitate tracheal intubation was ignored. The number of days free from ventilation and alive was the number of days with unsupported breathing for at least 24 sequential hours up to day 28, wherein patients that died were counted as having zero days free from ventilation. For the purpose of this study, a patient was considered to have been weaned from invasive ventilation if invasive ventilation was not restarted within the timeframe of the study.

### 2.5. Power Calculation

We did not perform a power calculation. The summed number of patients present in the databases of PRoVENT–COVID and PRoAcT-COVID served as the sample size.

### 2.6. Statistical Analysis

Continuous variables are presented as medians (first quartile–third quartile) and categorical variables as numbers and percentages. Groups were compared using the Mann–Whitney U test for continuous variables and the Fisher exact or Chi-square tests for categorical variables. Distribution plots were constructed to visualize the distributions of PEEP and FiO_2_.

To test the impact of the first and second wave on ICU and hospital discharge, competing risk analysis was used, with mortality as competing risk factor.

Covariate propensity score balancing was used to match patients between the two waves [13]. If the number of missing values was <5%, multiple imputation by the chained equation method (MICE) was performed. If the number of missing values per variable crossed 5%, patients with missing data were excluded from this part of the analysis. Based on clinical relevance, the following baseline characteristics were added to the logistic regression model to calculate the propensity score: body mass index, age, severity of ARDS, comorbidities including hypertension, heart failure, diabetes, chronic kidney disease, liver cirrhosis, chronic obstructive pulmonary disease, active hematological neoplasia, active solid neoplasia, neuromuscular disease, immunosuppression, and home medication including systemic glucocorticosteroids, inhalation glucocorticosteroids, angiotensin II receptor blocker, beta-blockers, insulin, statins, and calcium channel blockers. The nearest neighbor matching without replacement strategy was used for 1:1 matching of patients from the first to the second COVID-19 wave. An initial caliper width of 0.1 standard deviation of the logit of the propensity score was used to match the patients. If the covariates remained imbalanced after matching, lower caliper widths (until 0.01) were tested until balance was achieved. Variables’ standardized mean differences were visualized in LOVE plots and used to assess matching performance. We aimed for standardized mean differences of ≤0.1 (10%) [14]. The propensity score and study sites were added to the logistic and linear regression models to compare length of stay in intensive care unit and hospital, and ICU, hospital, and 28- and 90-day mortality between patients in the two consecutive waves.

As a relatively high incidence of NMBA use was found, a post hoc analysis comparing mortality in patients with and without NMBA was performed.

R version 4.2.2 (R foundation for Statistical Computing, Vienna, Austria) was used for this analysis, and a *p*-value of <0.05 was considered statistically significant.

## 3. Results

### 3.1. Patients

**Figure 1:**
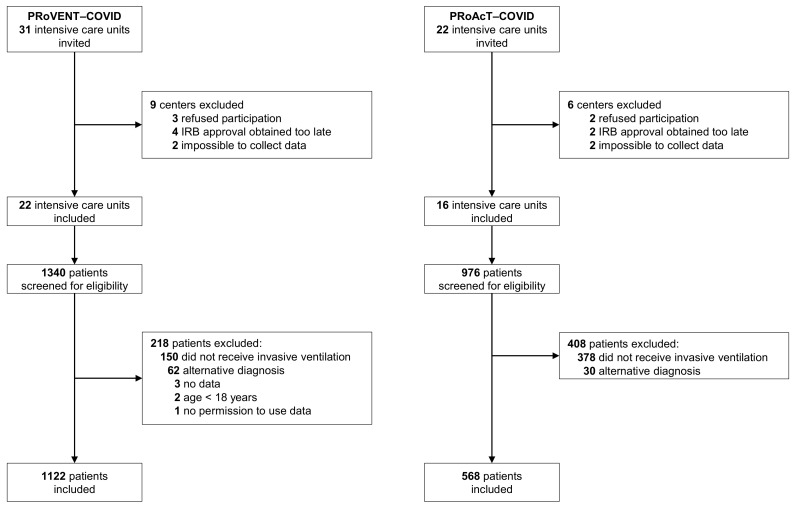
CONSORTs of PRoVENT–COVID and PRoAcT-COVID.

The databases of PRoVENT–COVID and PRoAcT-COVID contain data of 1122 patients from 22 ICUs and 976 patients from 16 ICUs, respectively (Figure 1). For the current analysis, we used all patients in the database of PRoVENT–COVID, but only 568 patients from the the database of PRoAcT-COVID. The main reason for exclusion of patients in the second study was not having received invasive ventilation. Compared to patients in the first wave, patients in the second wave were more often obese, had more often comorbidities such as diabetes, chronic kidney disease, or neuromuscular disease, and were treated more frequently with systemic steroids, beta blocking agents, or insulin at home (Table 1). Patients in the second wave were also sicker according to the SAPS II score and had worse oxygenation parameters on ICU admission. In both waves, nearly all patients had ARDS, with a higher incidence of severe ARDS in the second wave.

### 3.2. Unmatched Analysis

**Figure 2:**
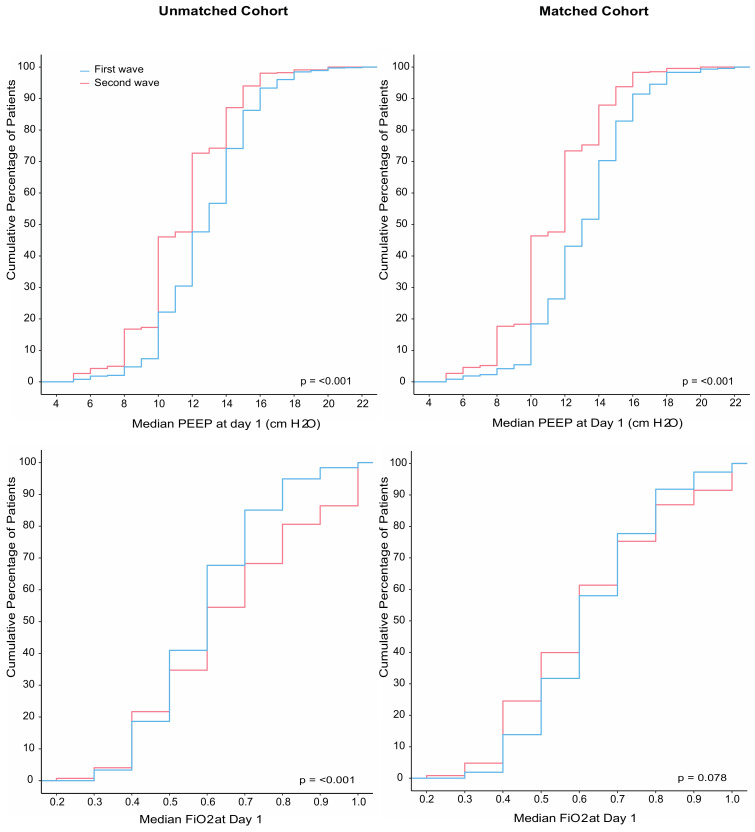
(**Left** panel) Cumulative distribution of positive end-expiratory pressure (PEEP) and fraction inspired oxygen (FiO_2_) on the first day of ventilation in the unmatched analysis; (**right** panel) cumulative distribution of positive end-expiratory pressure (PEEP) and fraction inspired oxygen (FiO_2_) on the first day of ventilation in the matched analysis.

**Figure 3:**
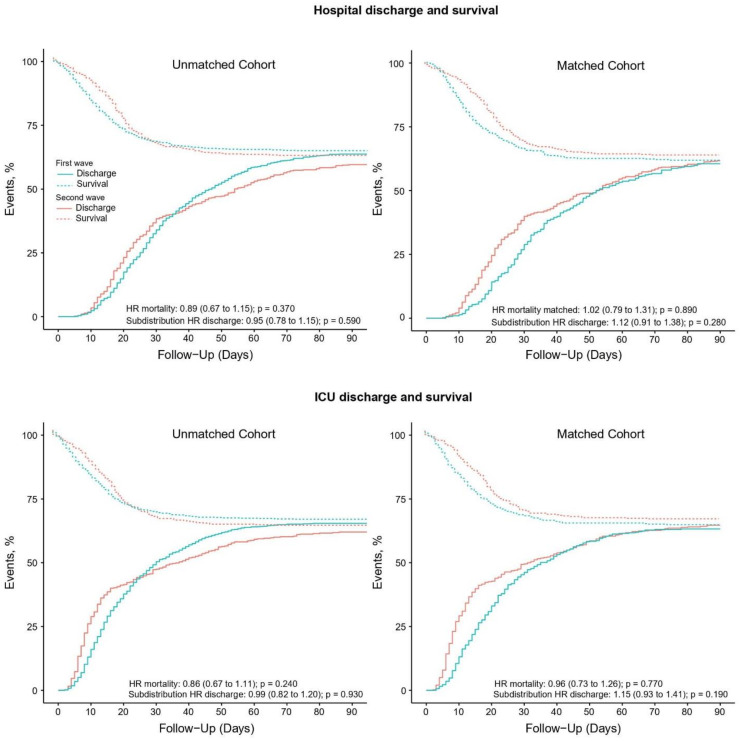
(**Left upper** panel) Competing risk analysis of hospital survival and discharge in the unmatched analysis; (**left lower** panel) competing risk analysis of ICU survival and discharge in the unmatched analysis; (**right upper** panel) competing risk analysis of hospital survival and discharge in the matched analysis; (**right lower** panel) competing risk analysis of ICU survival and discharge in the matched analysis.

Compared to patients in the first wave, patients in the second wave received pressure control and pressure support more often (Table 2). Patients in the second wave were ventilated with a lower median PEEP and higher median FiO_2_, and median SpO_2_ was lower (Figure 2). Prone positioning and ECMO were equally used. Neuromuscular blockade was used more often in the second wave. Patients in the second wave received a tracheostomy less often (Table 3). Duration of ventilation as well as ICU and hospital length of stay in survivors was shorter in the second wave. Mortality rates were similar in the two waves (Table 3 and Figure 3).

### 3.3. Matched Analysis

A total of 964 patients could be matched (Table S1, Figures S1–S5), with the baseline characteristics well balanced (Table 1). After propensity score matching, FiO_2_ was lower in the second wave (Table 2 and Figure 2). Differences in ICU and hospital LOS remained in the matched cohort but disappeared in multivariate models (Table 3 and Table S2). Mortality remained comparable between the two waves after matching (Table 3 and Table S3).

### 3.4. Post Hoc Analysis

**Figure 4:**
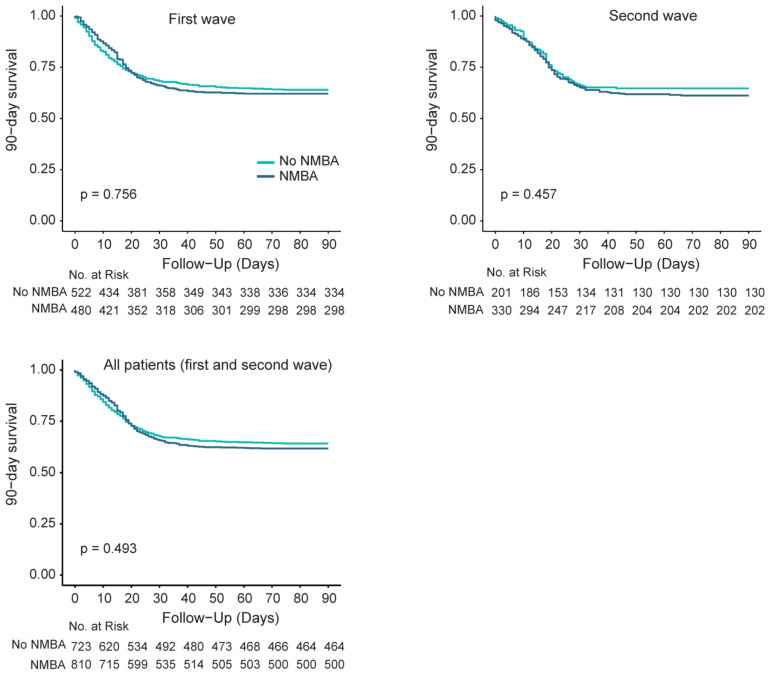
(**Left upper** panel) 90-day mortality between patients with and without NMBA in the first wave; (**right upper** panel) 90-day mortality between patients with and without NMBA in the second wave; (**left lower** panel) 90-day mortality between patients with and without NMBA in all patients, both from the first and second wave.

In both the first wave and second wave, and in all patients combined, no differences in 90-day mortality were found between patients who received NMBA and those who did not (Figure 4).

## 4. Discussion

The main findings of this post hoc analysis using the individual patient data of two consecutive studies performed in quick succession in the first year of the COVID-19 outbreak in the Netherlands can be summarized as follows: (i) compared to patients in the first wave, patients in the second wave received invasive ventilation, with lower median PEEP and higher median FiO_2_, and (ii) received neuromuscular blockage more often. Patients in the second wave (iii) received tracheostomy less often, (iv) were weaned earlier from invasive ventilation, and (v) had shorter lengths of stay, but (vi) had a similar mortality rate as patients in the first wave.

This study has several strengths. First, both academic and non-academic and teaching and non-teaching hospitals participated in the two parent studies. This allows for a realistic national representation of respiratory care and outcomes during the two COVID-19 waves. In addition, most hospitals that participated in the first study also participated in the second study, minimizing the risk of finding differences related to variations in pre-existing practice between hospitals. To ensure the high quality of the data obtained, all data collectors were trained and provided with clear instructions before capturing the granular data. The parent studies had no exclusion criteria, and for this current analysis, we only excluded patients receiving noninvasive ventilatory support from the second study. Follow-up was near complete in both studies, and the analysis strictly followed a predefined analysis plan.

The most important difference regarding ventilation practice was the lower median PEEP in the second wave. The best PEEP level in patients with ARDS due to COVID-19 remains uncertain [15]. This is similar to patients with ARDS due to another cause [16]. During the two waves, a policy-driven shift in the study population that received invasive ventilation occurred. Early in the pandemic, there was a scarcity of noninvasive ventilation and HFNO as well as uncertainty regarding whether these strategies could increase the risk of infection of the healthcare workers. This led to a policy of early intubation, a strategy that was followed by most hospitals during the first wave. In the second wave, caregivers were more reluctant to intubate patients early. This probably resulted in a selection of sicker patients in the second wave—patients with a lower lung compliance. Consequently, the used level of PEEP was expected to be higher in the second wave. Our findings, however, show a lower median PEEP in the second wave. Thus, caregivers had developed a preference for lower PEEP.

We previously described an association between the use of higher PEEP and worse outcomes in COVID-19 patients [17]. In that study, we showed that while higher PEEP improves oxygenation, it has an association with a longer duration of ventilation [17]. While this study was published after the second wave, i.e., after the second study in which we used individual patient data, it must be noted that the findings were previously presented and thus known soon after the first wave in various meetings in the Netherlands; it could be that this caused a change with respect to PEEP. Of interest, in the second wave, with use of lower PEEP, we found a shorter duration of ventilation, in line with the study mentioned above [17].

Prone positioning appeared to be an effective measure to improve oxygenation in COVID-19 patients [18]. In our analysis, the reported incidence of prone positioning is high in both waves, which is in line with earlier research [19,20].

The overall reported use of NMBA of 47.6% in the first wave and 62.3% in the second wave in the unmatched cohort is considerably high compared to earlier non–COVID-19 ARDS studies. For example, in the LUNG SAFE study, a trial containing data of 2377 ARDS patients in 50 different countries, the overall incidence of NMBA use was only 21.7% [21]. In COVID-19 ARDS patients, the reported use of NMBA varies from 25% to 84% [19,22,23], and in some countries, there was even a temporary shortage of NMBA [24]. This broad variability in NMBA use might be explained by international variation in critical care and differences in study populations. Indeed, we only included patients receiving invasive ventilation, while other trials also included patients requiring non-invasive ventilation [21,22]. Consequently, the use of NMBA in those studies is expected to be lower. In addition, the definition for NMBA use varies by study. In this analysis, both incidental and continuous NMBA administration were defined as NMBA use. In other studies, only continuous NMBA administration for a certain period of time was considered as NMBA use [21]. Given the relatively high incidence of NMBA use, we did perform a post hoc analysis, in which we found no differences in 90-day mortality between patients who did and did not receive NMBA. Guidelines discourage long-term use of NMBA [25,26], but the exact role of NMBA in critically ill COVID-19 patients remains a matter of debate. Of note, despite the guidelines, we found that the use of NMBA actually increased from the first to the second wave. This might be caused by the sicker population of patients in the second wave. Those patients are prone to deep saturation dips when coughing or when patient–ventilator asynchronies occur. This could justify the higher use of NMBA observed in the second wave.

We also found a lower incidence of tracheostomy in the second wave. This is in line with earlier research showing that early tracheostomy does not improve patient outcome [27,28]. This analysis showed a shorter duration of ventilation and a shorter length of hospital and ICU stay in survivors in the second wave. As suggested above, the use of lower PEEP might have contributed to this finding. The introduction of new effective treatments such as HFNO, steroids, and anti-inflammatory strategies as well as the pursuit of a less positive fluid balance after the first wave is also expected to have had a positive effect on the clinical outcomes in patients from the second wave [29,30,31]. Indeed, earlier research showed that those new therapies resulted in a shorter duration of ventilation, hospital and ICU stay, and mortality.

We did not find improved survival in the second wave. This is not in line with earlier research [2,32]. The positive effects of new insight and treatments on mortality rates in COVID-19 patients during the second wave might have been masked by the earlier mentioned differences in disease severity in this analysis. We did, however, find a shorter duration of ventilation and length of hospital and ICU stay in the second wave. These are not only important patient-centered outcomes but also important economic and societal results. A shorter duration of ventilation and hospitalization saves expenses, but it also creates medical capacity for other patients requiring medical care.

This analysis implies that ICU care of invasively ventilated COVID-19 patients quickly changed from the first to the second wave. During the outbreak of the first COVID-19 pandemic, there were many uncertainties regarding the optimal treatment and mechanical ventilation strategy for these patients. This resulted in the initiation of a tremendous amount of research in a short period of time to improve the clinical care of patients [33]. This new knowledge provided great insight into the epidemiology and characteristics of the disease as well as the effective treatment strategies for COVID-19 patients. Effective treatments were rapidly implemented into standard care, which is confirmed by this analysis.

This analysis has several limitations. Unfortunately, data on ICU admission criteria and ‘Do Not Resuscitate’ (DNR) codes were not collected in the PRoVENT–COVID and PRoAcT-COVID study. This is a limitation, since DNR codes might have led to ‘door selection’ and thereby could have interfered with our study population and findings. Only critically ill COVID-19 patients receiving invasive ventilation were included in this analysis. Thus, our analysis only provides insights into this selection of critically ill COVID-19 patients, which is another limitation. The retrospective design of the two parent studies is also a limitation, since it only enables us to speak of associations and not causality between characteristics, treatment, and outcomes. As only Dutch ICUs participated in the PRoVENT-COVID and PRoAcT-COVID studies, the results of this analysis might only be extrapolated to hospitals with health care systems similar to the Netherlands. Another limitation is that we are restricted to the variables collected in the two studies. For example, tidal volume and peak pressure were not collected in the PRoAcT-COVID study. Next to those variables, modifications of SARS-CoV-2 as well as the introduction of other unrecorded treatments such as steroids and anti-inflammatory strategies may have influenced our findings. However, since these changes were not captured in the datasets, we could not quantify their effect on our findings and thus could not correct for them in our analysis. Of note, vaccination status is not relevant to this analysis, as the COVID-19 vaccine was not available in the Netherlands until early after the second COVID-19 wave—indeed, the inclusion of patients in PRoAcT-COVID stopped several weeks before the national vaccination campaign started.

## 5. Conclusions

This analysis shows that important aspects of invasive ventilation changed over time. It also shows that outcomes improved between the two waves, manifested by a shortened length of stay, but similar mortality rates. This emphasizes the importance of continuous re-evaluation of patient characteristics and treatment during pandemics. Therefore, in future COVID-19 waves, it is critical to remain vigilant regarding recognizing changes in patient and disease characteristics as well as aspects of care. To capture these changes, scientific research remains essential. Our findings could also imply that caution is warranted in the default use of high PEEP. However, future research considering potential confounding factors is needed to confirm these findings.

## Figures and Tables

**Table 1:** Patient Characteristics and Medical History.

	Unmatched Analysis (*n* = 1690)			Matched Analysis (*n* = 964)		
	First Wave (*n* = 1122)	Second Wave (*n* = 568)	*p*	First Wave (*n* = 482)	Second Wave (*n* = 482)	*p*
Age, years	65.0 [57.0 to 72.0]	66.0 [58.0 to 73.0]	0.060	67.0 [59.0 to 73.0]	66.0 [58.0 to 72.0]	0.623
Sex, male	72.8 (817/1122)	73.9 (420/568)	0.642	83.0 (352/482)	74.3 (358/482)	0.715
Weight, kg	85.2 [78.0 to 96.0]	87.0 [78.0 to 100.0]	0.235	88.0 [80.0 to 98.1]	86.0 [77.6 to 99.0]	0.182
Height, cm	176.0 [170.0 to 183.0]	174.0 [168.0 to 180.0]	<0.001	175.0 [169.0 to 182.5]	174.0 [167.0 to 180.0]	0.008
BMI, kg/m^2^	27.7 [25.2 to 30.8]	28.9 [25.8 to 32.6]	<0.001	28.3 [26.1 to 31.6]	28.6 [25.7 to 32.3]	0.773
BMI groups *			<0.001			0.104
Underweight	0.3 (3/1101)	0.4 (2/562)		0.0 (0/475)	0.4 (2/477)	
Healthy Weight	22.4 (247/1101)	19.6 (110/562)		18.5 (88/475)	19.9 (95/477)	
Overweight	48.1 (530/1101)	38.4 (216/562)		45.9 (218/475)	39.4 (188/477)	
Obesity	29.2 (321/1101)	41.6 (234/562)		35.6 (169/475)	40.3 (192/477)	
SAPS II, *n* *	359	253		143	221	
SAPS II score	36.0 [29.0 to 44.0]	39.0 [32.0 to 45.0]	0.015	37.0 [30.0 to 45.0]	39.0 [32.0 to 45.0]	0.660
Severity of ARDS *			<0.001			0.983
No ARDS	3.3 (36/1102)	0.4 (2/561)		0.4 (2/482)	0.4 (2/482)	
Mild	17.1 (188/1102)	11.6 (65/561)		13.3 (64/482)	13.3 (64/482)	
Moderate	68.9 (759/1102)	58.3 (327/561)		67.4 (325/482)	66.6 (321/482)	
Severe	10.8 (119/1102)	29.8 (167/561)		18.9 (91/482)	19.7 (95/482)	
Comorbidities						
Arterial hypertension	33.9 (380/1122)	36.8 (209/568)	0.235	37/8 (182/482)	35.9 (173/482)	0.593
Heart failure	4.4 (49/1122)	4.9 (28/568)	0.622	6.2 (30/482)	4.6 (22/482)	0.318
Diabetes mellitus	22.3 (250/1122)	28.5 (162/568)	0.006	28.0 (135/482)	26.1 (126/482)	0.562
Kidney disease	4.2 (47/1122)	7.0 (40/568)	0.014	6.0 (29/482)	6.0 (29/482)	1.000
Liver cirrhosis	0.3 (3/1122)	0.5 (3/568)	0.410	0.6 (3/482)	0.4 (2/482)	1.000
COPD	7.8 (88/1122)	8.5 (48/568)	0.705	8.3 (40/482)	9.1 (44/482)	0.732
Hematological cancer	1.4 (16/1122)	2.5 (14/568)	0.171	1.0 (5/482)	1.5 (7/482)	0.773
Solid cancer	2.5 (28/1122)	3.7 (21/568)	0.170	3.3 (16/482)	3.5 (17/482)	1.000
Neuromuscular disease	0.7 (8/1122)	1.9 (11/568)	0.029	1.0 (5/482)	1.5 (7/482)	0.773
Immunosuppression	2.1 (24/1122)	2.6 (15/568)	0.498	3.1 (15/482)	2.3 (11/482)	0.552
Home medication						
Systemic glucocorticosteroids	3.4 (38/1122)	5.6 (32/568)	0.038	5.8 (28/482)	5.0 (24/482)	0.669
Inhalation glucocorticosteroids	11.1 (125/1122)	13.7 (78/568)	0.132	13.7 (66/482)	13.5 (65/482)	1.000
Angiotensin II receptor blocker	11.3 (127/1122)	14.4 (82/568)	0.072	14.9 (72/482)	12.4 (60/482)	0.303
Beta blockers	18.8 (211/1122)	25.0 (142/568)	0.004	21.8 (105/482)	23.7 (114/482)	0.539
Insulin	7.0 (78/1122)	10.7 (61/568)	0.009	8.7 (42/482)	8.7 (42/482)	1.000
Statins	29.4 (330/1122)	32.7 (186/568)	0.163	31.1 (150/482)	32.8 (158/482)	0.629
Calcium channel blockers	17.6 (197/1122)	16.2 (92/568)	0.495	17.2 (83/482)	16.8 (81/482)	0.932
Laboratory results						
Creatinine, μmol/L	75 [62 to 98]	79 [62 to 109]	0.051	77.0 [63.0 to 102.0]	78.0 [62.0 to 108.2]	0.816
PaO_2_/FiO_2_ ratio	152 [120 to 192]	125.0 [95 to 167]	<0.001	140.5 [109.0 to 177.8]	133.6 [105.4 to 175.0]	0.120

Data are presented as median [IQR] or % (*n*/*n*). * In a selection of patients, BMI, SAPS II and ARDS severity could not be collected.

**Table 2:** Ventilation management and support treatments.

	Unmatched Analysis (*n* = 1690)			Matched Analysis (*n* = 964)		
	First Wave (*n* = 1122)	Second Wave (*n* = 568)	*p*	First Wave (*n* = 482)	Second Wave (*n* = 482)	*p*
PEEP, cm H_2_O *	13 [11 to 15]	12 [10 to 14]	<0.001	13.0 [11.0 to 15.0]	12.0 [10.0 to 13.0]	<0.001
PEEP ranges *			<0.001			<0.001
PEEP 5–10 cm H_2_O (*n*/*n*)	22.2 (247/1114)	46.0 (261/567)		18.4 (88/478)	46.4 (223/481)	
PEEP 11–15 cm H_2_O (*n*/*n*)	64.1 (714/1114)	48.0 (272/567)		64.4 (308/478)	47.4 (228/481)	
PEEP >15 cm H_2_O (*n*/*n*)	13.7 (153/1114)	6.0 (34/567)		17.2 (82/478)	6.2 (30/481)	
FiO_2_ *	0.6 [0.5 to 0.7]	0.6 [0.5 to 0.8]	<0.001	0.6 [0.5 to 0.7]	0.6 [0.5 to 0.7]	0.078
FiO_2_ ranges *			0.017			0.005
FiO_2_ 0.2–0.4 (*n*/*n*)	3.3 (37/1111)	4.1 (23/567)		1.9 (9/476)	4.8 (23/481)	
FiO_2_ 0.4–0.6 (*n*/*n*)	37.6 (418/1111)	30.7 (174/567)		29.8 (142/476)	35.1 (169/481)	
FiO_2_ ≥ 0.6 (*n*/*n*)	59.0 (656/1111)	65.3 (370/567)		68.3 (325/476)	60.1 (289/481)	
SpO_2_ *, %	95 [93 to 96]	94 [91 to 96]	<0.001	94.0 [93.0 to 96.0]	94.0 [92.0 to 96.0]	0.001
SpO_2_ ranges *			<0.001			<0.001
SpO_2_ 95–100% (*n*/*n*)	52.6 (588/1118)	39.8 (220/553)		48.5 (233/480)	42.4 (199/469)	
SpO_2_ 90–95% (*n*/*n*)	44.2 (494/1118)	47.6 (263/553)		47.9 (230/480)	48.0 (225/469)	
SpO_2_ < 90% (*n*/*n*)	3.2 (36/1118)	12.7 (70/553)		3.5 (17/480	9.6 (45/469)	
Ventilation mode *			<0.001			<0.001
Volume controlled (*n*/*n*)	20.8 (219/1053)	19.4 (110/567)		20.5 (91/444)	18.9 (91/481)	
Pressure controlled (*n*/*n*)	59.1 (622/1053)	64.0 (363/567)		57.2 (254/444)	64.4 (310/481)	
Pressure support (*n*/*n*)	5.4 (57/1053)	10.9 (62/567)		5.2 (23/444)	11.4 (55/481)	
INTELLIVENT–ASV (*n*/*n*)	4.2 (44/1053)	4.1 (23/567)		5.6 (25/444)	3.5 (17/481)	
Other modes (*n*/*n*)	10.5 (111/1053)	1.6 (9/567)		11.5 (51/444)	1.7 (8/481)	
Prone positioning, % (*n*/*n*)^#^	56.2 (625/1113)	59.8 (308/515)	0.178	60.2 (289/480)	57.7 (254/440)	0.461
ECMO, % (*n*/*n*) ^#^	1.1 (12/1107)	0.4 (2/480)	0.251	1.1 (5/476)	0.5 (2/409)	0.461
Neuromuscular blockade, % (*n*/*n*) ^#^	47.6 (534/1122)	62.3 (354/568)	<0.001	47.9 (231/482)	60.6 (292/482)	<0.001

Data are presented as median [IQR] or % (*n*/*n*). * First day of invasive ventilation; ^#^ first 4 days of invasive ventilation.

**Table 3:** Clinical outcomes.

	Unmatched Analysis (*n* = 1690)			Matched Analysis (*n* = 964)		
	First Wave (*n* = 1122)	Second Wave (*n* = 568)	*p*	First Wave (*n* = 482)	Second Wave (*n* = 482)	*p*
Tracheostomy ^&^, % (*n*/*n*)	17.1 (190/1112)	11.3 (64/568)	0.002	18.1 (87/480)	11.2 (54/482)	0.003
Duration of ventilation						
Duration of ventilation, days	14.0 [8.0 to 23.0]	11.0 [6.0 to 23.0]	0.001	15.0 [9.0 to 24.2]	11.0 [6.0 to 23.0]	<0.001
Days free of ventilation and alive at day 28, days ^%^	2.0 [0.0 to 16.0] (1065/1122)	0.0 [0.0 to 21.0] (516/568)	0.017	0.0 [0.0 to 14.0] (453/482)	0.0 [0.0 to 21.0] (439/482)	<0.001
Days free of ventilation at day 28, deceased patients excluded	12.0 [0.0 to 18.0] (743/1122)	18 [0.0 to 23.0] (338/568)	<0.001	11.0 [0.0 to 17.0] (306/482)	18.0 [0.0 to 23.0] (295/482)	<0.001
LOS						
ICU, days	15.0 [9.0 to 26.0]	14.0 [8.0 to 26.0]	0.105	16.0 [9.0 to 27.0]	13.0 [8.0 to 26.0]	0.034
ICU, in survivors, days	17.0 [10.0 to 29.0]	12.0 [7.0 to 29.0]	<0.001	20.0 [11.0 to 32.0]	12.0 [7.0 to 29.0]	<0.001
Hospital, days	23.0 [14.0 to 37.0]	22.0 [15.5 to 34.0]	0.616	25.0 [13.0 to 38.3]	22.0 [15.0 to 36.0]	0.399
Hospital, in survivors, days	29.0 [20.0 to 44.0]	25.0 [17.0 to 45.8]	0.013	32.0 [22.0 to 48.0]	24.5 [17.0 to 45.0]	<0.001
Mortality						
28-day mortality, %	28.9 (318/1102)	32.1(176/549)	0.190	30.7 (146/476)	30.5 (142/465)	1.000
90-day mortality, %	37.7 (383/1015)	37.5 (199/531)	0.956	40.0 (175/438)	35.6 (159/447)	0.188
ICU mortality, %	32.6 (356/1091)	34.6 (190/549)	0.437	35.8 (167/466)	32.9 (153/465)	0.370
Hospital mortality, %	35.9 (367/1022)	36.3 (198/545)	0.869	38.6 (169/438)	34.3 (158/461)	0.188

Data are presented in median [IQR] or % (*n*/*n*). ^&^ First 28 days of invasive ventilation. ^%^ The number of days free from ventilation and alive, wherein patients that died were counted as having zero days free from ventilation.

## Data Availability

The datasets used and/or analyzed during the current study are available from the authors on reasonable request.

## Supplementary Materials

The following supporting information can be downloaded at: https://www.mdpi.com/article/10.3390/jcm12134507/s1, Figure S1: Distribution of missing data; Figure S2: Strip plot distribution imputed data (magenta) and observed data (blue); Figure S3: Histogram matched and unmatched cohort; Figure S4: Bal plot matched and unmatched cohort; Figure S5: Love plot matched and unmatched cohort; Table S1: Missing data for propensity score matching; Table S2: Multivariate model for length of hospital and ICU stay; Table S3: Multivariate model for mortality.

## Abbreviations

ARDS	Acute Respiratory Distress Syndrome
BMI	Body Mass Index
COPD	Chronic Obstructive Pulmonary Disease
COVID-19	Coronavirus disease 2019
DNR	Do Not Resuscitate
ECMO	Extra Corporal Membrane Oxygenation
FiO_2_	Fraction Inspired Oxygen
ICU	Intensive Care Unit
NMBA	Neuromuscular Blocking Agent
PEEP	Positive End-Expiratory Pressure
PRoVENT–COVID	PRactice of VENTilation in Patients with Novel Coronavirus disease
PRoAcT-COVID	Practice of adjunctive treatments in critically ill COVID-19 patients
SAPS	Simplified Acute Physiology Score
SPO_2_	Oxygen Saturation

## Appendix A

PRoVENT–COVID investigators:

S. Ahuja; J.P. van Akkeren; A.G. Algera; C.K. Algoe; R.B. van Amstel; P. van de Berg; D.C. Bergmans; D.I. van den Bersselaar; F.A. Bertens; A.J. Bindels; J.S. Breel; C.L. Bruna; M.M. de Boer; S. den Boer; L.S. Boers; M. Bogerd; L.D. Bos; M. Botta; O.L. Baur; H. de Bruin; L.A. Buiteman-Kruizinga; O. Cremer; R.M. Determann; W. Dieperink; J. v. Dijk; D.A. Dongelmans; T.P. Dormans; M.J. de Graaff; M.S. Galekaldridge; L.A. Hagens; J.J. Haringman; S.T. van der Heide; P.L. van der Heiden; l.l. Hoeijmakers; L. Hol; M. W. Hollmann; J. Horn; R. van der Horst; E.L. Ie; D. Ivanov; N.P. Juffermans; E. Kho; E.S. de Klerk; A.W. Koopman; M. Koopmans; S. Kucukcelebi; M.A. Kuiper; D.W. de Lange; I. Martin–Loeches; G. Mazzinari; D.M van Meenen; N. van Mourik; S.G. Nijbroek; E.A. Oostdijk; F. Paulus; C.J. Pennartz; J. Pillay; I.M. Purmer; T.C. Rettig; O. Roca; J.P. Roozeman; M.J. Schultz; A. Serpa Neto; C. Sivakorn; G.S. Shrestha; M.E. Sleeswijk; P.E. Spronk; A.C. Strang; W. Stilma; P. Swart; A. Tri; A.M. Tsonas; C.M.A. Valk; A.P. Vlaar; L.I. Veldhuis; W.H. van der Ven; P. van Velzen; P. van Vliet; P. van der Voort; L. van Welie; B. van Wijk; T. Winters; W.Y. Wong; A.R. van Zanten.

PRoAcT-COVID investigators:

E. Aydeniz; P. van de Berg; D.C. Bergmans; M. Bevers; S. den Boer; L.S. Boers; L.D. Bos; M. Botta; L.A. Buiteman-Kruizinga; W. Coene; M. Delmte; Vincenzo Di Leo; D.A. Dongelmans; T.P. Dormans; L.M. Elting; A.A. Esmeijer; M. Gama de Abreu; A.R. Girbes; M.J. de Graaff; D.M. Go; R.L. Goossen; H.J. Hansen; J.J. Haringman; L. Hol; M.W. Hollmann; P.L. van der Heiden; J. Horn; L.E. van Ingen; N.P. Juffermans; M.A. Kuiper; L.J. Kuipers; E. Koornstra; A. Lokhorst; S.G. Nijbroek; I. Martin–Loeches; G. Mazzinari; S. Myatra; F. Paulus; M. Offermans; T. Pisters; A. Prins; P. van Oosten; J. Pillay; I.M. Purmer; A.S. Rezaee; T.C.D. Rettig; O. Roca; N.M. Rosenberg; N. Schavemaker; A.A. Sciascera, M.J. Schultz; A. Serpa Neto; M.E. Sleeswijk; G. Sherstha; W. Stilma; A.C. Strang; P.E. Spronk; P.R. Tuinman; A.M. Tsonas; C.M.A. Valk; M. Verboom; A.P. Vlaar; F.L. van der Ven; W. H. van der Ven; P. van Velzen; E.J. Verhoef; T.D. Vermeulen; P. van Vliet; J.J. J.S. Voorham; P.H. van der Voort; M. van der Woude; Weiner; N. Yaali; J.M. Zandvliet; A.R. van Zanten; T.Z. van Zijl; S.A. Zonneveld, I. Martin–Loeches (Dublin, Ireland).
