# Supplementary material for: Differences in Ventilation Management and Outcomes between the Two First Waves of the COVID-19 Pandemic—A Comparison between Two Nationwide Observational Studies in The Netherlands
Source: J Clin Med. 2023 Jul 5;12(13):4507. doi: 10.3390/jcm12134507 (PMC10342245; doi:10.3390/jcm12134507)
Supplement: Supplementary file 1 [file jcm-12-04507-s001.zip › jcm-2361907-supplementary.pdf]

Supplementary Materials

Liselotte Hol [1,*], Marcus J. Schultz [2,3,4], Ignacio Martin-Loeches [5,6], David M. P. van Meenen [1,2], Ary Serpa Neto [2,7,8] and Frederique Paulus [2,9] on behalf of the PRoVENT–COVID [†,‡] and PRoAcT-COVID Investigators [‡,§]

1 Department of Anesthesiology, Amsterdam University Medical Centers, Location AMC, 1105 AZ Amsterdam, The Netherlands
2 Department of Intensive Care, Amsterdam University Medical Centers, Location AMC, 1105 AZ Amsterdam, The Netherlands
3 Mahidol Oxford Tropical Medicine Research Unit (MORU), Mahidol University, Bangkok 10400, Thailand
4 Nuffield Department of Medicine, University of Oxford, Oxford OX37BN, UK
5 Department of Intensive Care Medicine, Multidisciplinary Intensive Care Research Organization (MICRO), St James's Street, Leinster, D08 NYH1 Dublin, Ireland
6 Department of Clinical Medicine, Trinity College, D02 PN40 Dublin, Ireland
7 Department of Critical Care Medicine, Hospital Israelite Albert Einstein, Sao Paolo 05652-900, Brazil
8 Department of Critical Care Medicine, Austin Hospital and University of Melbourne, Melbourne 3084, Australia
9 Centre of Applied Research, Faculty of Health, Amsterdam University of Applied Sciences, 1091 GC Amsterdam, The Netherlands
* Correspondence: l.hol@amsterdamumc.nl
† PRoVENT–COVID, Practice of Ventilation in COVID-19.
‡ Full list of investigators is provided in Appendix A.
§ PRoAcT-COVID, Practice of Adjunctive Treatments in intensive care unit patients with COVID-19.

TABLE OF CONTENTS

Page 3 **Table S1** Missing data for propensity score matching

Page 4 **Figure S1** Distribution of missing data

Page 5 **Figure S2** Stripplot distribution imputed data (magneta) and observed data (blue)

Page 6 **Table S2** Multivariate model for length of hospital and ICU stay

Page 7 **Table S3** Multivariate model for mortality

Page 8 **Figure S3** Histogram matched and unmatched cohort. Caliper set at 0.02.

Page 9 **Figure S4** Bal plot matched and unmatched cohort. Caliper set at 0.02.

Page 10 **Figure S5** Love plot matched and unmatched cohort. Caliper set at 0.02.

Table S1. Amount of Missing Data.

	Unmatched N=1690	First wave N=1122	Second Wave N=568
BMI, kg/m^2	27 (1.6)	21 (1.9)	6 (1.1)
Severity of ARDS	27 (1.6)	20 (1.8)	7 (1.2)
Comorbidities			
Arterial hypertension	0 (0.0)	0 (0.0)	0 (0.0)
Heart failure	0 (0.0)	0 (0.0)	0 (0.0)
Diabetes mellitus	0 (0.0)	0 (0.0)	0 (0.0)
Kidney disease	0 (0.0)	0 (0.0)	0 (0.0)
Liver cirrhosis	0 (0.0)	0 (0.0)	0 (0.0)
COPD	0 (0.0)	0 (0.0)	0 (0.0)
Hematological cancer	0 (0.0)	0 (0.0)	0 (0.0)
Solid cancer	0 (0.0)	0 (0.0)	0 (0.0)
Neuromuscular disease	0 (0.0)	0 (0.0)	0 (0.0)
Immunosuppression	0 (0.0)	0 (0.0)	0 (0.0)
Home medication			
Systemic glucocorticosteroids	0 (0.0)	0 (0.0)	0 (0.0)
Inhalation glucocorticosteroids	0 (0.0)	0 (0.0)	0 (0.0)
Angiotensin II receptor blocker	0 (0.0)	0 (0.0)	0 (0.0)
Beta blockers	0 (0.0)	0 (0.0)	0 (0.0)
Insulin	0 (0.0)	0 (0.0)	0 (0.0)
Statins	0 (0.0)	0 (0.0)	0 (0.0)
Calcium channel blockers	0 (0.0)	0 (0.0)	0 (0.0)

Data presented as number (%). Multiple imputation conducted using predictive mean matching for BMI and proportional odds model for severity of ARDS, 20 iteratoins and 5 databases.

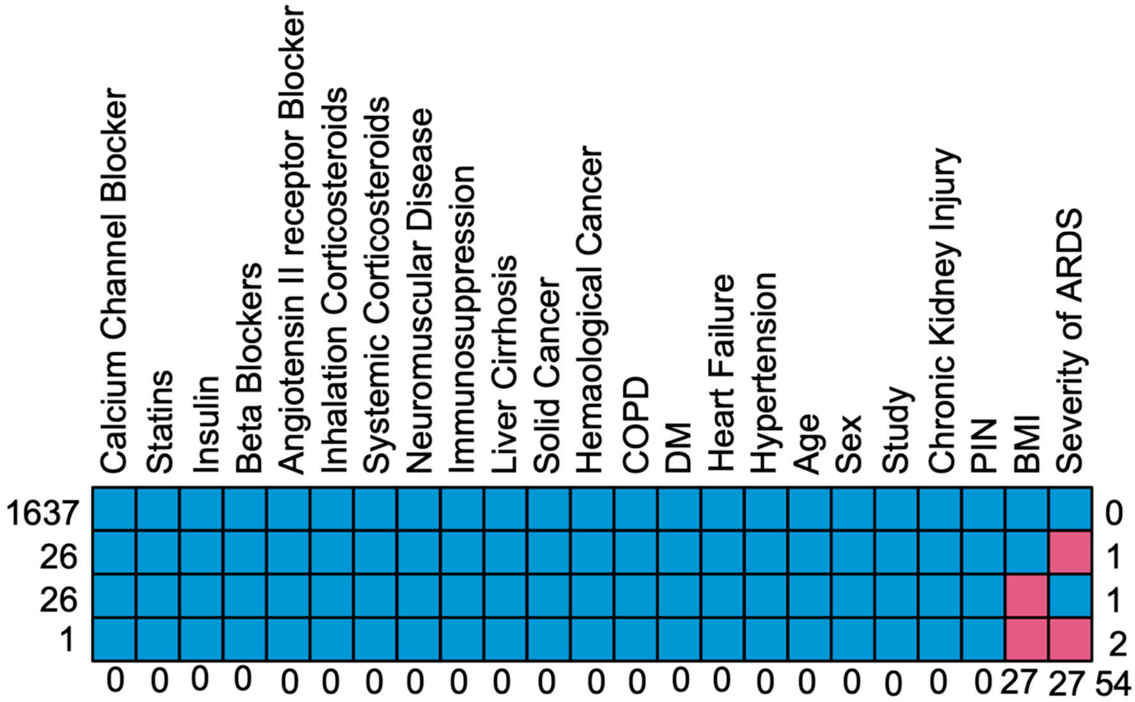

Figure S1. Distribution of missing data.

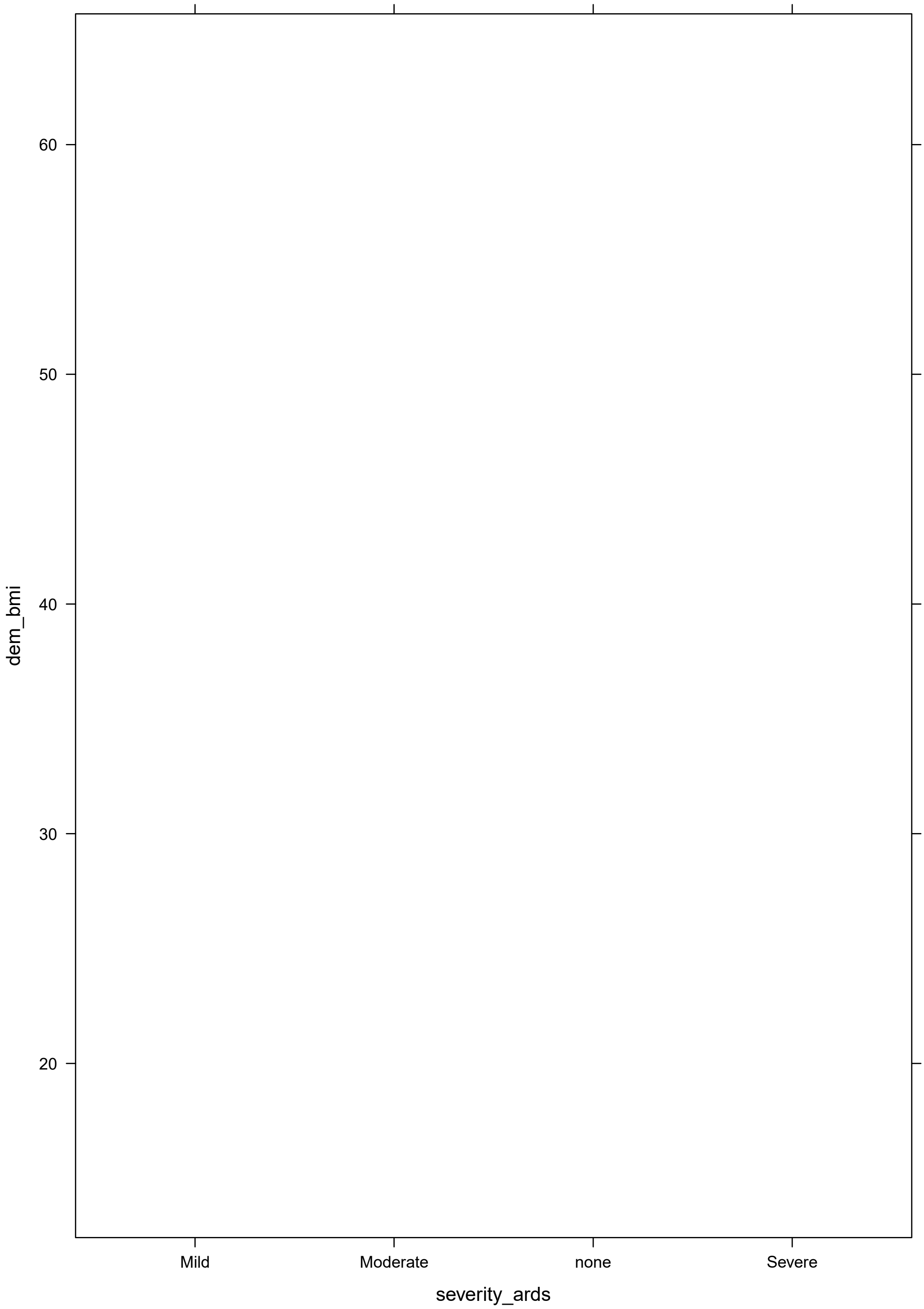

Figure S2. Stripplot distribution imputed data (magneta) and observed data (blue).

Table S2. Multivariate model for length of hospital and length of ICU stay in matched cohort**.**

	β-coefficient (95% CI)	*P*
Length of hospital stay		
First COVID–19 outbreak	Reference	
Second COVID–19 outbreak	-0.004 (-3.114 to 3.151)	0.998
Length of hospital stay in survivors		
First COVID–19 outbreak	Reference	
Second COVID–19 outbreak	-2.852 (-6.869 to 1.268)	0.168
Length of ICU stay		
First COVID–19 outbreak	Reference	
Second COVID–19 outbreak	-0.251 (-2.805 to 2.327)	0.848
Length of ICU stay in survivors		
First COVID–19 outbreak	Reference	
Second COVID–19 outbreak	-2.795 (-6.217 to 0.673)	0.112

Models are adjusted for the covariate balancing propensity score. Centers are entered as random effect.

Table S3. Multivariate model for mortality in matched cohort.

	Effect estimate (95% CI)	*P*
28–day mortality		
First COVID–19 outbreak	Reference	
Second COVID–19 outbreak	HR 0.899 (0.681 to 1.187)	0.450
90–day mortality		
First COVID–19 outbreak	Reference	
Second COVID–19 outbreak	HR 0.849 (0.654 to 1.102)	0.220
Overall ICU–mortality		
First COVID–19 outbreak	Reference	
Second COVID–19 outbreak	OR 0.862 (0.619 to 1.199)	0.378
Overall hospital mortality		
First COVID–19 outbreak	Reference	
Second COVID–19 outbreak	OR 0.845 (0.606 1.177)	0.319

Models are adjusted for the covariate balancing propensity score. Centers are entered as random effect.
HR: Hazard Ratio; OR: Odds Ratio.

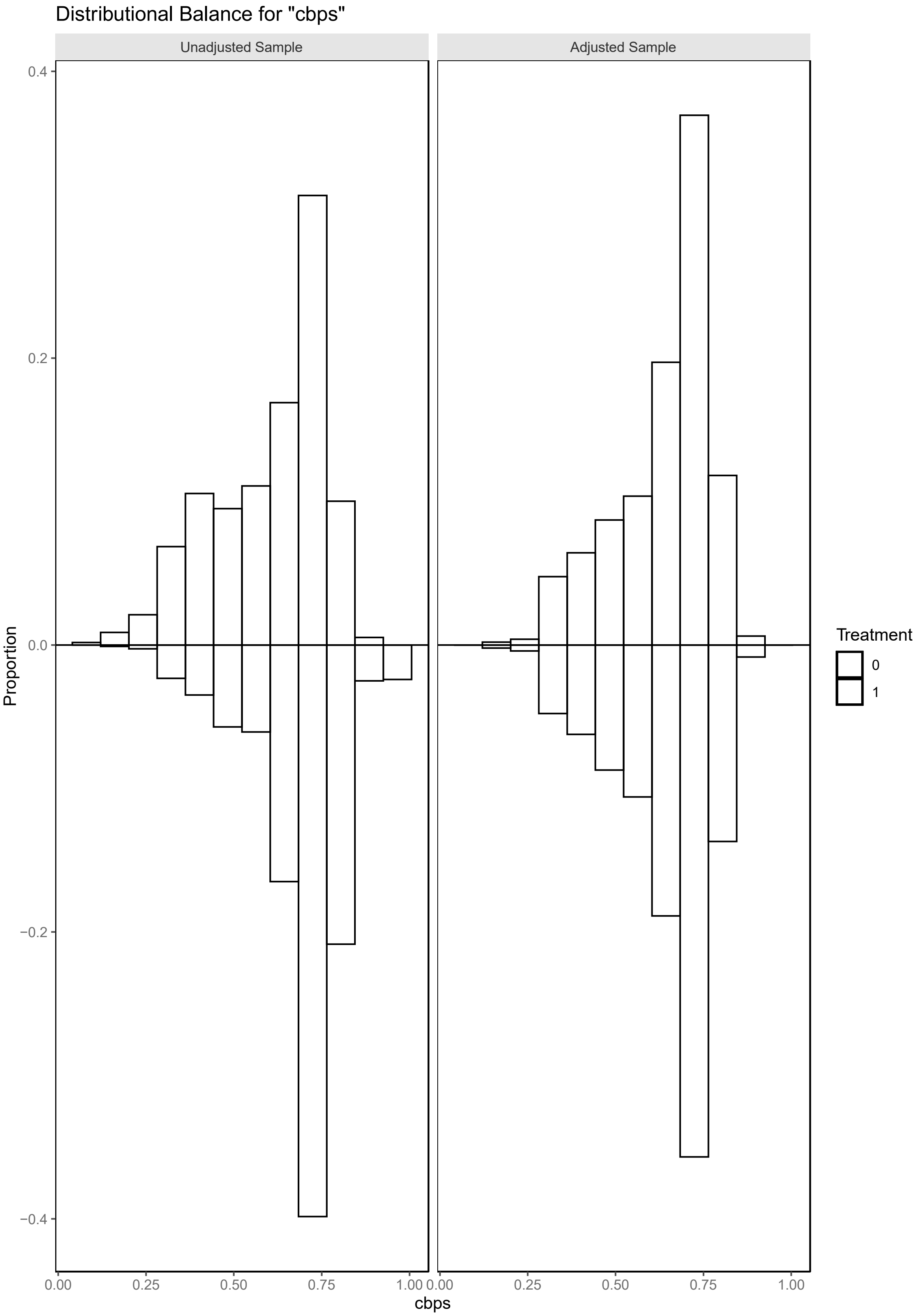

Figure S3. Histogram matched and unmatched cohort. Caliper set at 0.02.

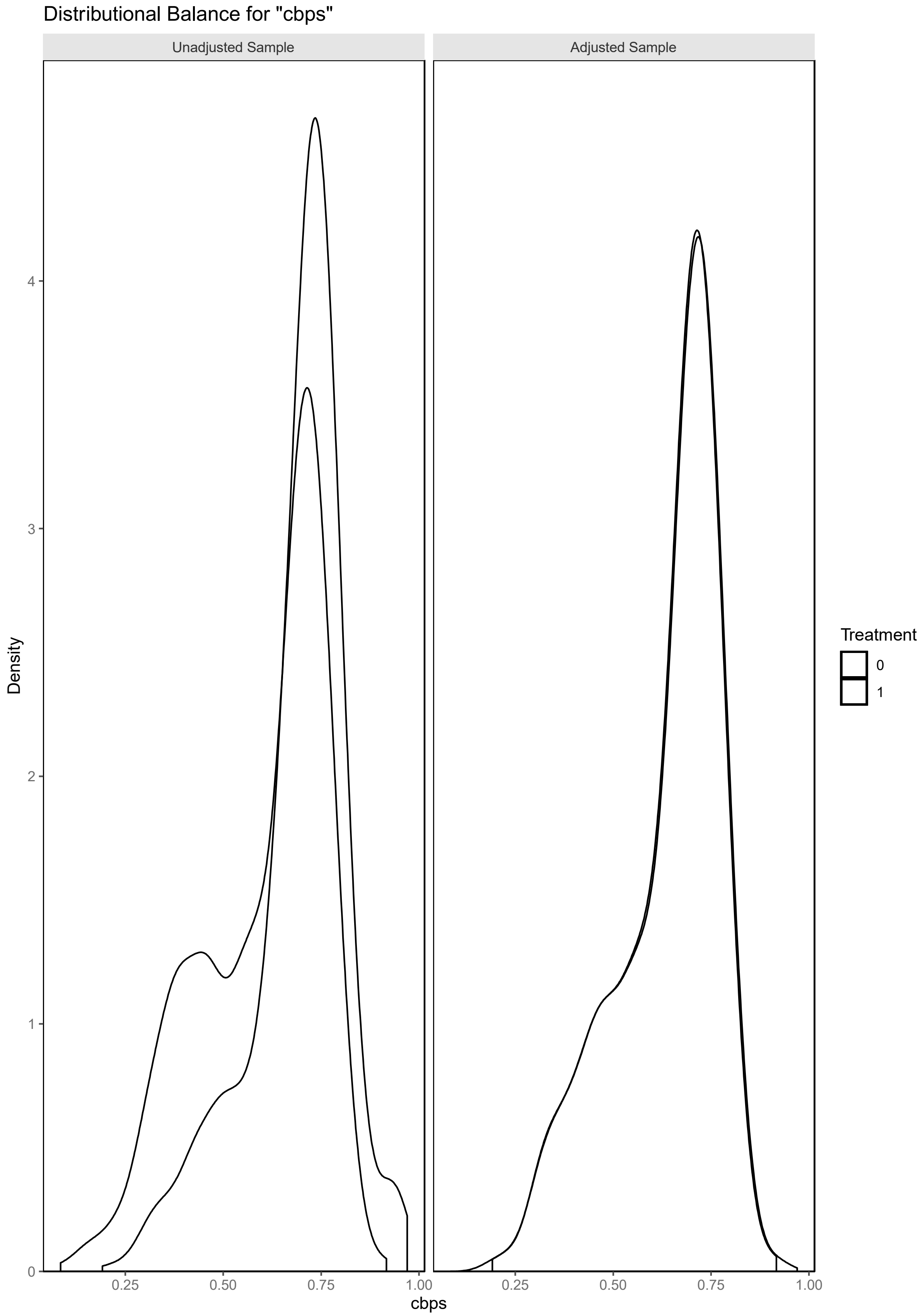

Figure S4. Bal plot matched and unmatched cohort. Caliper set at 0.02.

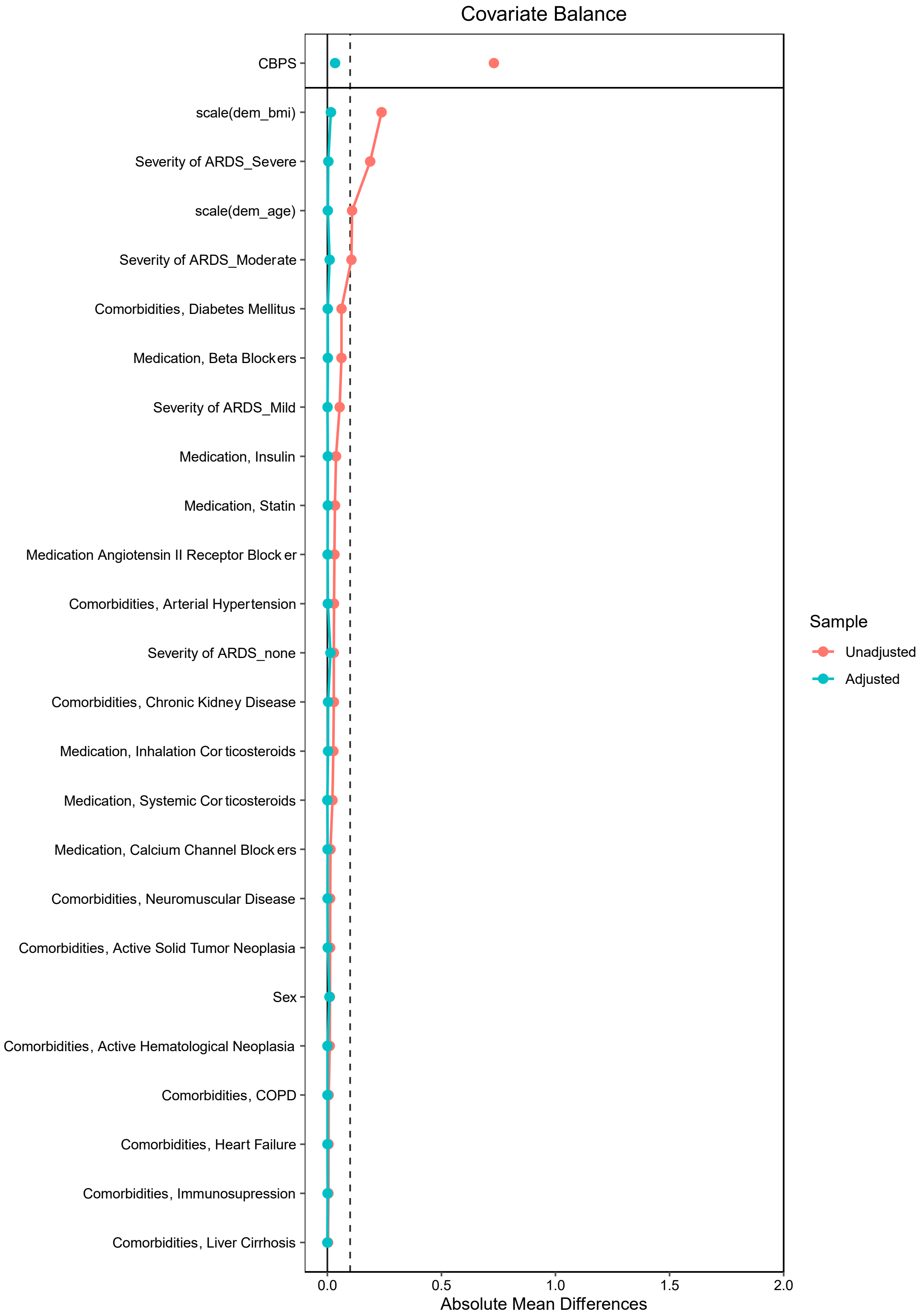

Figure S5 Love plot matched and unmatched cohort. Caliper set at 0.02.
